# 376. Sensitivity and Specificity of the WHO Probable SARS-CoV-2 Case Definition Among Symptomatic Healthcare Personnel

**DOI:** 10.1093/ofid/ofab466.577

**Published:** 2021-12-04

**Authors:** Han Nguyen, Sarah Weber, Yachana Kataria, Manisha Cole, Elizabeth Duffy, Elizabeth Ragan, Jacquelyn Turcinovic, Nancy Miller, William P Hanage, John Connor, Cassandra Pierre, Karen Jacobson, Sara Lodi, Tara Bouton

**Affiliations:** 1 Boston University School of Public Health, Boston, Massachusetts; 2 Boston Medical Center, Boston, Massachusetts; 3 Boston University, Boston, Massachusetts; 4 Harvard T.H. Chan School of Public Health, Boston, MA

## Abstract

**Background:**

SARS-CoV-2 continues to spread globally, including in limited resource settings. It is therefore important to derive general case definitions that can be useful and accurate in the absence of timely test results. We aim to validate the World Health Organization (WHO) case definition, a symptom-screening tool currently used to identify SARS-CoV-2 cases in a cohort of symptomatic health care providers (HCP) who completed a symptom survey interview and received a PCR test at Boston Medical Center (BMC) between March 13, 2020 and May 5, 2020.

**Methods:**

We classified each HCP as a probable or not probable case of SARS-CoV-2 based on the WHO case definition. Using PCR test as gold standard, we computed the sensitivity and specificity of the WHO case definition. We used a stepwise logistic regression model on all PCR-tested HCP to identify symptoms predictive of PCR positivity.

**Results:**

Of 328 included HCP, 109 (33.2%) were PCR positive, 213 (64.9%) negative, and 6 (1.8%) had indeterminate test result. The sensitivity and specificity of the WHO case definition were 65.1% and 74.6%, respectively. The positive predictive value was 56.8% and the negative predictive value was 80.7%. Symptoms found to be predictive of PCR positivity were fever, headache, loss of smell and/or loss of taste, and muscle ache/joint pain. Sore throat was found to be predictive of PCR negativity. The area under the curve using the final model was 0.8412. All statistically significant symptoms included in the final model, were also included in the WHO case definition.

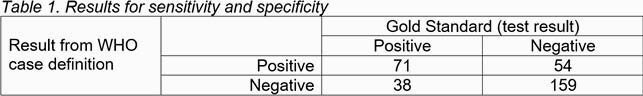

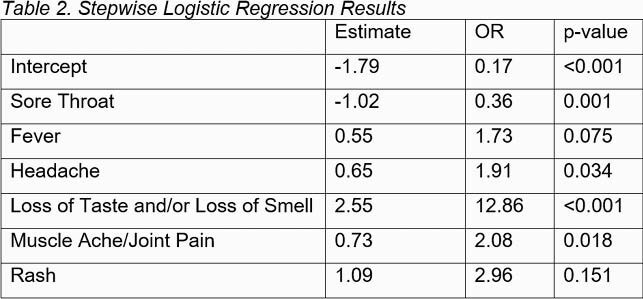

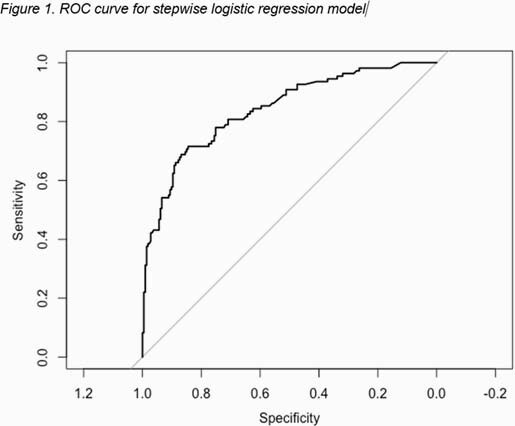

**Conclusion:**

In our largely symptomatic HCP cohort, our model yielded similar symptoms to those identified in the WHO probable case definition. As seen in similar studies, it is unlikely that further adjustment will improve the performance of a SARS-CoV-2 case definition. However, it is concerning that 35% (38/109) of PCR positive SARS-CoV-2 HCP would have been classified as not probable cases by the WHO definition, given that this definition does not even include asymptomatic cases. This is further evidence for global building of laboratory capacity and development of affordable diagnostics to improve global pandemic control.

**Disclosures:**

**All Authors**: No reported disclosures

